# Supplementary material for: Biomechanical Behaviour and Biocompatibility of Ureidopyrimidinone-Polycarbonate Electrospun and Polypropylene Meshes in a Hernia Repair in Rabbits
Source: Materials (Basel). 2019 Apr 10;12(7):1174. doi: 10.3390/ma12071174 (PMC6480159; doi:10.3390/ma12071174)
Supplement: Supplementary file 1 [file materials-12-01174-s001.pdf]

materials

Article

Biomechanical Behaviour and Biocompatibility of Ureidopyrimidinone-Polycarbonate Electrospun and Polypropylene Meshes in a Hernia Repair in Rabbits

Marina Gabriela M. C. Mori da Cunha [1,2], Lucie Hympanova [1,2,3], Rita Rynkevic [1,2,4], Tristan Mes [5], Anton W. Bosman [5] and Jan Deprest [1,2,6,*]

1 Centre for Surgical Technologies, Group Biomedical Sciences, KU Leuven, 3000 Leuven, Belgium; biamori@gmail.com (M.G.M.C.M.d.C.); lucie.hympanova@upmd.eu (L.H.); r.rynkevic@gmail.com (R.R.)
2 Department of Development and Regeneration, Woman and Child, Group Biomedical Sciences, KU Leuven, 3000 Leuven, Belgium
3 Institute for the Care of Mother and Child, Third Faculty of Medicine, Charles University, 14700 Prague, Czech Republic
4 INEGI, Faculdade de Engenharia da Universidade do Porto, Universidade do Porto, 4099-002 Porto, Portugal;
5 SupraPolix BV, 5611 Eindhoven, The Netherlands; mes@suprapolix.com (T.M.); bosman@suprapolix.com (A.W.B.)
6 Pelvic Floor Unit, University Hospitals KU Leuven, 3000 Leuven, Belgium
* Correspondence: Jan.Deprest@uzleuven.be; Tel.: 32-1634-4215; Fax: 32-1634-4205

Supplementary Material and Methods

Supplement S1—Mechanical Testing of the Meshes

The equipment used for testing is developed at INEGI Biomechanics Laboratory. The machine has four perpendicular aluminum alloy arms, connected to four actuators and two load cells (with a 50N capacity). A preload of 0.1 N was applied to remove the slack from the sample, using a constant elongation rate of 5 mm/min. The clamp-to-clamp distance and width were estimated using a digital camera. A constant elongation rate of 10 mm/min was used to load the specimen.

Supplement S2—Anesthesia and Analgesia

Animals were premedicated with ketamine (10mg/kg, Nimatek, Eurovet Animal Health B.V., Bladel, Netherlands) and midazolam (3mg/kg, Mylan, Hoeilaart, Belgium). Further, they were anesthetized briefly using 2% isoflurane (Iso-Vet®; Piramal Healthcare UK Ltd.; Morpeth; UK) in 100% oxygen so that an epidural anesthesia could be given using a mixture of lidocaine hydrochloride 2%-adrenaline (2.5 mg/kg, Xylocaine, AstraZeneca, Zoetermeer, The Netherlands), bupivacaine hydrochloride 0.5% (0.5 mg/kg, Marcaine, AstraZeneca) and morphine (0.1 mg/kg, Sterop Laboratoria, Brussels, Belgium). Animals were additionally to epidural kept in 0.5% of isoflurane in 100% oxygen. A Ringer lactate solution (10 mL/kg/h) was used as perfusion fluid. Rabbits were operated under sterile conditions and kept on a heating pad until recovery.

Postoperatively, pain relief was done by buprenorphine (0.03 mg/kg, Vetergesic®, Alstoe, York, UK) for three days.

Supplement S3—Immunohistochemistry and Morphometric Analysis

Sections were then heated at 98 °C for 1 h in citrate buffer (10 mmol/L, pH 6) to enhance antigen retrieval. After blocking unspecific binding with blocking buffer (bovine serum albumin 2%, non-fat dry milk 1%, Tween 80 0.1%) for one hour at room temperature, slides were incubated with primary

antibody overnight at 4 °C. Specific secondary antibodies were used. Negative controls included buffer alone. Specific labelling was detected with EnVision™/HRP Detection Kit (Dako). The colour reaction was developed with 3,3'-diaminobenzidine (Sigma) and sections were counterstained with Mayer haematoxylin. Sections were then dehydrated through graded ethanol, cleared in xylene, and mounted in dePex BDH (VWR International, Haasrode, Belgium).

Supplementary figure

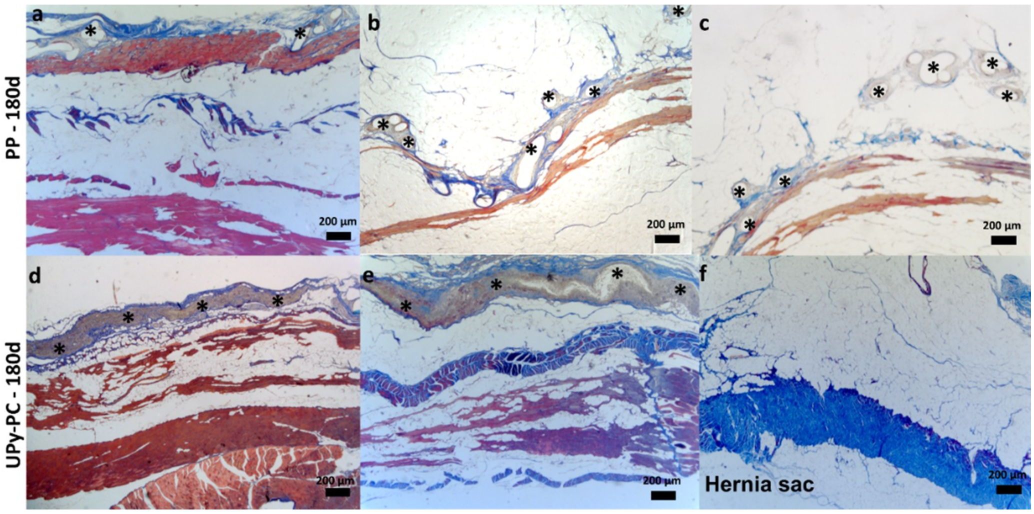

Figure S1. Representative images of Masson´s Trichrome staining (50× magnification) of the abdominal wall of rabbits after implantation of polypropylene (**a**–**c**) and electrospun UPy-PC (**d**–**f**) at 180 days in the gap bridging model. Images show moderate (**a**,**d**) to severe (**b**,**c**,**e**) levels of muscle atrophy of the abdominal wall in non-herniated samples. Only a thick layer of connective tissue can be observed in the abdominal wall of a herniated sample (**f**).

© 2019 by the authors. Submitted for possible open access publication under the terms and conditions of the Creative Commons Attribution (CC BY) license (http://creativecommons.org/licenses/by/4.0/).
